# Selective Amplification of Plasmonic Sensor Signal for Cortisol Detection Using Gold Nanoparticles

**DOI:** 10.3390/bios12070482

**Published:** 2022-07-01

**Authors:** Gaye Ezgi Yılmaz, Yeşeren Saylan, Ilgım Göktürk, Fatma Yılmaz, Adil Denizli

**Affiliations:** 1 Department of Chemistry, Hacettepe University, Ankara 06800, Turkey; gayeyilmaz@hacettepe.edu.tr (G.E.Y.); yeseren@hacettepe.edu.tr (Y.S.); ilgim@hacettepe.edu.tr (I.G.); 2 Department of Chemistry Technology, Bolu Abant Izzet Baysal University, Bolu 14900, Turkey; yilmaz_f@ibu.edu.tr

**Keywords:** cortisol detection, gold nanoparticles, molecular imprinting, plasmonic sensor

## Abstract

Herein, gold nanoparticles (AuNP)-modified cortisol-imprinted (AuNP-MIP) plasmonic sensor was developed for signal amplification and real-time cortisol determination in both aqueous and complex solutions. Firstly, the sensor surfaces were modified with 3-(trimethoxylyl)propyl methacrylate and then pre-complex was prepared using the functional monomer N-methacryloyl-L-histidine methyl ester. The monomer solution was made ready for polymerization by adding 2-hydroxyethyl methacrylate to ethylene glycol dimethacrylate. In order to confirm the signal enhancing effect of AuNP, only cortisol-imprinted (MIP) plasmonic sensor was prepared without AuNP. To determine the selectivity efficiency of the imprinting process, the non-imprinted (AuNP-NIP) plasmonic sensor was also prepared without cortisol. The characterization studies of the sensors were performed with atomic force microscopy and contact angle measurements. The kinetic analysis of the AuNP-MIP plasmonic sensor exhibited a high correlation coefficient (R^2^ = 0.97) for a wide range (0.01–100 ppb) with a low detection limit (0.0087 ppb) for cortisol detection. Moreover, the high imprinting efficiency (k′ = 9.67) of the AuNP-MIP plasmonic sensor was determined by comparison with the AuNP-NIP plasmonic sensor. All kinetic results were validated and confirmed by HPLC.

## 1. Introduction

Cortisol is one of the most important glucocorticoids synthesized in the zona fasciculate of the adrenal cortex. It is released in response to physiological stress and has various effects on different functions in the body. It can also affect almost every organ system such as nervous, immune, cardiovascular, respiratory, reproductive, musculoskeletal, and covering systems [1]. Furthermore, it is an important biomarker for numerous diseases and plays a significant role in the regulation of physiological processes such as carbohydrate metabolism, blood pressure, glucose levels, and also in homeostasis of the cardiovascular, renal, endocrine, skeletal, and immune systems. Excess cortisol in the body can cause obesity, fatigue, bone fragility, and Cushing’s syndrome and cortisol deficiency can cause Addison’s disease with symptoms of fatigue, excessive weight loss, and darkening of skin folds [2]. Elevations in cortisol levels can lead to brain aging, cognitive damage, sarcopenia, a decreased bone mineral density, and an impaired immune system and the disruption of cortisol levels is often accompanied by the severity of other illnesses such as COVID-19 [3,4,5,6]. For all of these reasons, the development of an effective, sensitive, and selective method for the detection of cortisol has great importance.

The conventional clinical assessment of cortisol levels is performed using a variety of test methods, including radioimmunoassays, chromatographic techniques, flow immunoassays, fluorimetric tests, and enzyme-linked immunosorbent tests [7,8,9]. Today, cutting-edge measurement methods such as quartz crystal microbalance, Raman spectroscopy, impedance spectroscopy, and amperometric detection have been used to determine cortisol levels. Although these analytical methods allow the detection of cortisol within a range of normal physiological variations, they are limited by the inability to detect cortisol effectively due to their long detection time, low sensitivity, multiple steps, lack of specificity, and procedural complexity [10].

Sensors are promising analytical tools that provide a high selectivity and sensitivity and can also be miniaturized and fully automated. They are categorized into three main groups such as optical, electrochemical, and mass [11]. Optical sensors have great advantages over traditional analytical techniques as they enable direct, label-free, and real-time detection of a large number of chemical and biological substances. Their advantages are a high specificity, cost-effectiveness, and sensitivity [12]. They are also fast, reproducible, and easy-to-use analytical tools [13]. One of the most commonly used optical sensor sub-classes is surface plasmon resonance (SPR). The SPR phenomenon measures the change in optical reflectivity of a thin metal film such as gold or silver that arises due to changes in the refractive index at the metal surface [14]. SPR sensors have a wide range of applications in many fields such as disease diagnosis, disease monitoring, immunoassays, clinical analysis, pharmaceuticals, industrial processing, monitoring, veterinary, environmental pollution control, food, and agricultural applications [15]. Compared to classical analytical methods, SPR sensors have advantages such as real-time monitoring, a low cost, a fast analysis time, and a high sensitivity [16]. The application of sensor surface modifications can improve the selectivity performance against target molecules. Surface plasmon resonance (SPR) binding analysis methodology is used to study molecular interactions [17]. The level of cortisol in saliva was studied using a six-channel portable SPR sensor due to its quick, real-time, and label-free sensing capabilities by Steven et al. [18].

Recently, the application of noble metal-based nanoparticles (NP) such as gold (Au) and silver (Ag) can increase the sensitivity of sensors due to their ability to enhance responses [19]. AuNP with a diameter of 1–100 nm have a high surface energy and surface-to-volume ratio to ensure stable immobility of a large number of molecules that retain their activity. AuNP provides direct and fast electron transfer between a wide variety of electroactive species and electrode materials, as well as being used as signal amplification tags in many different types of sensors, thanks to its light-emitting properties and the fairly large enhancement capacity of the local electromagnetic field [20]. For medical applications, AuNP offers a versatile functionalization possibility by targeting molecules, allowing for more precise diagnostics and localized therapeutic effects [21]. Furthermore, researchers detected cortisol levels using AuNP-based long-range SPR sensors, grating-based fiber optic sensors, and lossy mode resonance-based SPR sensors [22,23,24,25]. The plasmonic sensors integrated with molecularly imprinted nanoparticles have received great attention as biological recognition elements [26]. Molecular imprinting is a process applied to create recognition sites in a macromolecular matrix using a template molecule [27]. Molecularly imprinted polymers have many superior properties such as stable chemical, physical, and mechanical properties, the ability to withstand a high pressure and a high temperature, a strong resistance to acids and alkalis, an easy synthesis, a long-term performance life, reusability, and recycling [28].

Herein, a selective amplification of plasmonic sensor signals for cortisol detection was obtained using gold nanoparticles. After the preparation and characterization steps, kinetic studies were performed with plasmonic sensors. The cortisol samples were prepared at different concentrations and interacted with the plasmonic sensors. Then, the binding kinetic parameters were calculated, and the sensing performance was investigated. In addition, selectivity, reusability, and complex sample analysis spiked with cortisol were performed. Finally, the plasmonic sensor detection ability was validated by HPLC analyses.

## 2. Experimental Studies

### 2.1. Chemical Materials and Optical System

L-histidine methyl ester and methacryloyl chloride used in the synthesis of N-methacryloyl-L-histidine methyl ester (MAH) monomer. Sodium citrate tribasic dehydrate, gold (III) chloride trihydrate, 3-(trimethoxylyl)propyl methacrylate (TMSPM), 2-hydroxyethyl methacrylate (HEMA), azoisobisbutyronitrile (AIBN), and ethylene glycol dimethacrylate (EGDMA) were obtained from Sigma. Cortisol was purchased from Merck. Plasmonic sensor surfaces were purchased from GenOptics. SPR imager II (GWC Technologies) device was used for sensing. The sensor surfaces had 1 mm × 18 mm × 18 mm of dimensions and 50 nm of gold thickness. The laser light source of the system was Quartz Halogen Lamps (6 volts 10 watts 2000 H). SF10 equilateral prism was used.

### 2.2. Synthesis and Characterization of Functional Monomer and Gold Nanoparticles

Synthesis of MAH was carried out by applying a procedure performed formerly [29]. Briefly, 5 g of L-histidine methyl ester and 0.2 g of hydroquinone were dissolved in methylene chloride solution. A total of 12.7 g of trimethylamine was added to this solution and 5.0 mL of methacryl chloride was poured into this solution. This solution was stirred at room temperature for 2 h. At the end of the reaction, unreacted methacryl chloride was extracted with 10% NaOH. The aqueous phase was evaporated, and the residue (MAH) was dissolved in ethanol.

Fourier transform infrared (FTIR) spectroscopy was used for functional group analysis of MAH, MAH-AuNP, MAH-AuNP-cortisol pre-complexes, and also cortisol. The FTIR spectra of the samples were obtained separately with placing samples at the slot of the device and the total amounts of transmittance were measured in the wavenumber range of 400–4000 cm^−1^. AuNP was prepared by reduction of HAuCl_4_ salt to Au using sodium citrate according to the Turkevich method [30]. A dynamic light scattering (DLS) analysis was conducted to estimate the hydrodynamic size of the AuNP by the Nano Zetasizer Instrument (NanoS, Malvern Instruments). In the analysis, the density of deionized water was 0.88 mPa.s and the refractive index was adjusted to 1.33. The solution of nanoparticles was placed in the sample chamber of the analyzer and size analysis was performed. The number of nanoparticles was calculated by measuring the light scattering at room temperature at an incidence angle of 90° and the measurements were repeated three times. The average gold core diameter (D) was reported for each sample by averaging the sizes obtained from the transmission electron microscopy (TEM) image. The diameter of at least 100 nanoparticles was determined from TEM image to characterize the size distribution and the standard deviation was calculated for each nanoparticle sample by averaging these nanoparticles. The TEM image of the AuNP sample was obtained using a 300 keV/FEG transmission electron microscope (FEI Tecnai-G2-F30). After determining the average diameter of the AuNP, the concentration of AuNP was computed as follows [31]:

(1) $$N=\frac{\pi }{6}\frac{{\rho D}^{3}}{M}$$

(2) $$C=\frac{N_{\mathrm{total}}}{{\mathrm{NVN}}_{A}}$$

Equation (1) was used to calculate the number of Au atoms per nanoparticle (N) and Equation (2) was used to calculate AuNP concentration (C). In the equation, M corresponds to the atomic weight of Au, ρ to the density of Au, D to the diameter of AuNP, N_A_ to Avogadro’s number, and V to the solution volume.

### 2.3. Modification and Preparation of Plasmonic Sensors

The modification step was performed by direct attachment of vinyl group onto gold chip surface as shown in Figure S1A of Supplementary Materials [32]. TMSPM solution in methyl alcohol (50:50, *v*/*v*) was added to the chip surface and kept at 35 °C for 24 h. Then, in order to remove the unbound TMSPM monomer, the surface of the plasmonic sensor was washed with ethyl alcohol. For the preparation of the AuNP-MIP plasmonic sensor, firstly the MAH-AuNP complex was formed by coordinating AuNP with the functional monomer. Then, MAH-AuNP complex was mixed with cortisol to prepare the MAH-AuNP-cortisol pre-complex. For this step, 0.4 mmol of MAH monomer was incubated with 0.01 nmol of AuNP in a rotator at 20 rpm for 1 h. Then, 0.05 mmol of cortisol was mixed with the MAH-AuNP complex on a rotator at 20 rpm for 1 h to form the MAH-AuNP-cortisol pre-complex as seen in Figure S1B. After that, 0.4 mmol of EGDMA crosslinker was added to the pre-complex mixture and 0.2 mmol of HEMA monomer was mixed to provide hydrophilicity. A total of 2.5 mg of AIBN was added to the monomer mixture as an initiator, and 10 μL of mixture was dropped onto the modified sensor surface. A spin-coating device was used to distribute the mixture homogeneously on the sensor surface. The polymerization reaction was carried out for 1 h under a UV lamp (365 nm, 100 W) to form an imprinted polymer on the surface. The cortisol was removed from the surface using a mixture of desorption solution (methyl alcohol and acetic acid, *v*/*v*, 1:1). For this purpose, the plasmonic sensor was washed in 20 mL of desorption solution in a shaking incubator at 90 rpm and room temperature for 2 h. Afterwards, the desorbed sensor surface was washed in a mixture of deionized water and ethyl alcohol and dried in an oven (40 °C). MIP plasmonic sensor was fabricated by the procedure used for the preparation of the AuNP-MIP plasmonic sensor without the addition of AuNP. Besides, AuNP-NIP plasmonic sensor was prepared by the procedure used for the preparation of the AuNP-MIP plasmonic sensor except for the addition of cortisol.

### 2.4. Characterization of Plasmonic Sensors

The roughness and depth characterization of bare, AuNP-MIP, MIP, and AuNP-NIP plasmonic sensor surfaces were investigated by atomic force microscopy (AFM) analyses. Asylum Research 3D Standalone device was used to measure the needle tip, which was thinned down to atomic dimensions and sharpened. Two and three-dimensional images were obtained by scanning the surface of the plasmonic sensors with high resolution. The images of the surfaces were taken in half-touch mode by placing them in a diameter with millimeter dimensions in air. While the scanning speed of the images was 1 μm/s, an image was obtained from an area of 1 × 1 μm^2^. In order to determine the hydrophilicity of AuNP-MIP, MIP, and AuNP-NIP plasmonic sensor surfaces, contact angle values measuring wettability were determined by KRUSS DSA100 device. During the process, the contact angle values were obtained by the sessile drop method and the wettability was measured by dropping of water on the surfaces. After dropping water to three different regions, images were taken in each region and different contact angles were determined. FTIR spectroscopy was used for the analysis of functional groups of AuNP-MIP, MIP, and AuNP-NIP plasmonic sensors. The FTIR spectra of the plasmonic sensors were obtained separately with placing samples at the slot of the device and the total amount of transmittance were measured in the wavenumber range of 400–4000 cm^−1^.

### 2.5. Kinetic Analyses with Plasmonic Sensors

SPR imager II device was used to display the surface plasmon curves obtained as a result of kinetic analyses of AuNP-MIP, MIP, and AuNP-NIP plasmonic sensors. When the flow of solution containing cortisol began, the polarized light reached the surface of the plasmonic sensors and the angle of reflected light of the plasmonic sensors was changed during image acquisition in order to determine the %change of refractive index (ΔR) of the light. The ΔR values obtained against the incidence angle of the light for each sample were plotted and reported. Kinetic analyses on samples at different cortisol concentrations were made using SPR device. Firstly, cortisol-containing samples were prepared in the range of 0.01–100 ppb and applied to AuNP-MIP plasmonic sensor. PBS-ethyl alcohol solution (50:50, *v*/*v*) was used as an equilibrium solution. After setting an optimum resonance angle, the determined refraction angle was settled as the working angle. SPR view software was used for the kinetic analyses. After the resonance angle was determined, the analyses were carried out by following the equilibrium-adsorption-desorption steps for each concentration. The equilibrium solution was first passed through the sensor system and adjusted to the resonance angle for 180 s. After the system reached equilibrium, cortisol solutions were applied to the sensor system through a peristaltic pump at a flow rate of 0.2 mL/min for 300 s, respectively. The observed shift values in the resonance frequency were reported instantaneously. After observing the change in angle, desorption solution was applied to the system for 120 s. Following the completion of the analysis, the deionized water passed to the system for 10 min and the system was brought to equilibrium solution. These processes were also repeated for each sample applications.

### 2.6. Selectivity and Reusability Analyses

In order to indicate the selectivity of the AuNP-MIP plasmonic sensor against the cortisol, the adsorption studies were evaluated by performing with competitive agents fluticasone (100 ppb) and clobetasol (100 ppb) in the adsorption solution. Equilibrium-adsorption-desorption cycles followed in the competitive analyses were carried out in the same way. In order to determine the imprinting efficiency of the AuNP-MIP plasmonic sensor and the effects of AuNP on the signal response, the MIP and AuNP-NIP plasmonic sensors were fabricated and cortisol solutions (100 ppb) applied to each plasmonic sensors. The selectivity (k) and relative selectivity (k′) coefficients were calculated according to Equations (3) and (4).

(3) $$k=∆R_{\mathrm{cortisol}}/∆R_{\mathrm{competitor}}$$

(4) $$k\prime =k_{\mathrm{MIP}}/k_{\mathrm{NIP}}$$

In order to estimate the reusability of the AuNP-MIP plasmonic sensor, a 100 ppb cortisol sample was prepared in the equilibrium solution and interacted with the sensor in five repetitions. Firstly, adsorption solution was applied to the system for 180 s and the system was brought to the equilibrium. Then, cortisol sample and desorption solution were given to the system one after the other at every 180 s interval. The real-time signal response was observed by performing five repeats of the steps including these processes.

### 2.7. Complex Environment Analyses

In order to evaluate whether AuNP-MIP plasmonic sensor could be used in complex environment or not, artificial plasma and artificial urine samples were prepared and applied to the sensor system. For this purpose, the artificial plasma and artificial urine samples were prepared by spiking cortisol samples at a concentration of 100 ppb in the equilibrium solution and interacted with the AuNP-MIP plasmonic sensor.

### 2.8. Validation Analyses

The detection of the cortisol in the samples applied to the AuNP-MIP plasmonic sensor was confirmed using the HPLC system (Dionex, Ultimate HPLC). Cortisol sample solutions of 1–100 ppb were used to create the calibration curve. While preparing the solutions, they were prepared from stock cortisol solution at a concentration of 1 mg/mL in 10 mM methyl alcohol:acetonitrile:water (21:25:54, *v*/*v*) as a buffer solution. Prepared buffer solutions were used as mobile phase in HPLC system. Cortisol samples were prepared with artificial plasma and artificial urine at a concentration of 100 ppb. Analyses of the prepared samples were carried out at a wavelength of 220 nm using a C18 column [33].

## 3. Results and Discussion

### 3.1. Characterization of Functional Monomer and Gold Nanoparticles

**Figure 1:**
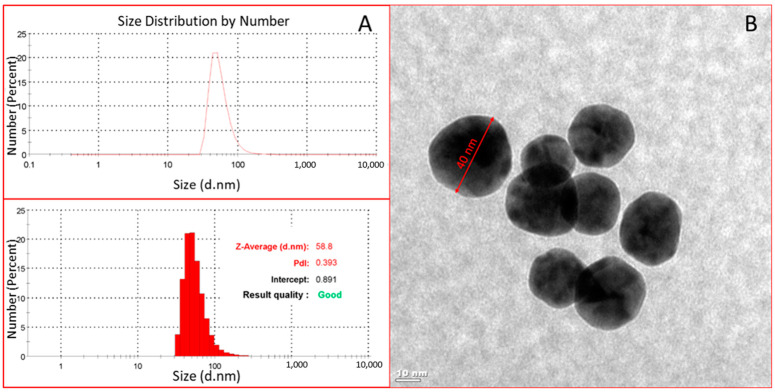
DLS analysis (**A**) and TEM image (**B**) of AuNP to obtain size distribution and shape.

When Figure S2 was examined, the wavenumber of the peak belonging to the C=O bond at 1732 cm^−1^ in the FTIR spectrum of the MAH monomer shifted from 1732 cm^−1^ to 1741 cm^−1^ after AuNP binding, and this indicated that AuNPs were coordinated with MAH monomer thoroughly. In the spectrum of the MAH-AuNP-cortisol pre-complex, the C-O bond peak was observed at a 1369 cm^−1^ wavelength and the peak of the C-N bond appeared at a 1292 cm^−1^ wavelength implying that cortisol was integrated into the structure. In order to detect the concentration of AuNP added to increase the plasmonic signal response, the size distribution analysis of AuNP was first performed with the DLS analysis. As seen in Figure 1, the polydispersity index value (PdI = 0.393) showed that the spheroidal AuNP with a moderately dispersed size distribution were obtained, and no aggregation was observed due to the absence of nanoparticles of very different sizes. The average size of AuNP was determined as 58.8 nm by measurements repeated three times. The concentration of AuNP were approximately measured by TEM image of AuNP. The estimated diameter was 41.14 ± 1.31 nm. After determining the diameter, the concentration of AuNP was computed as 2.0 × 10^−8^ M.

### 3.2. Characterization of Plasmonic Sensor Surfaces

**Figure 2:**
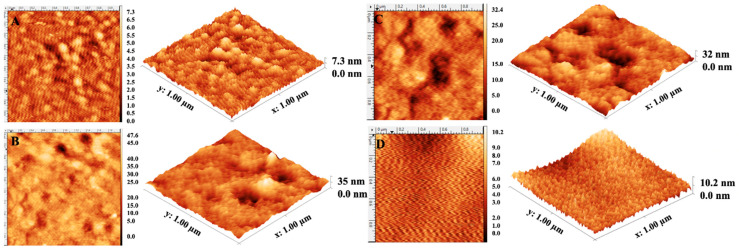
Two- and three-dimensional AFM images of the bare (**A**), AuNP-MIP (**B**), MIP (**C**), and AuNP-NIP (**D**) plasmonic sensors to show surface topographies.

Morphological properties of bare, AuNP-MIP, MIP, and AuNP-NIP plasmonic sensor surfaces were investigated by AFM analyses. The average roughness values were measured as 0.8 ± 0.2 nm, 4.1 ± 1.1 nm, 3.7 ± 2.3 nm, and 2.4 ± 1.9 nm, respectively (Figure 2). Also, the average depth values of bare, AuNP-MIP, MIP, and AuNP-NIP plasmonic sensor surfaces were measured as 3.4 nm, 16.59 nm, 14.87 nm, and 12.36 nm, respectively. These results demonstrated the successful and homogeneous binding of nanofilms to the gold surfaces of plasmonic sensors.

**Figure 3:**
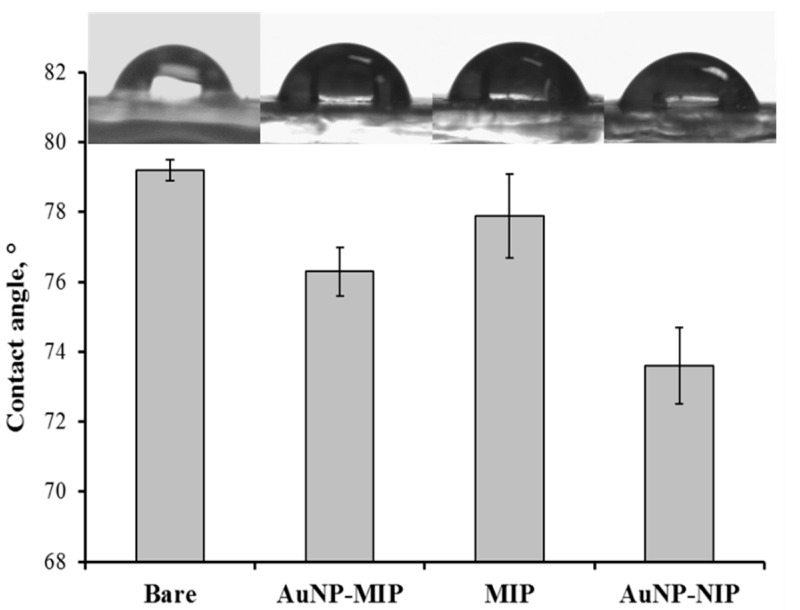
Contact angle values and images of bare, AuNP-MIP, MIP, AuNP-NIP sensors to depict hydrophilicity.

The contact angles of bare, AuNP-MIP, MIP, and AuNP-NIP plasmonic sensor surfaces were measured. As shown in Figure 3, the contact angle value of bare surface was 79.2° ± 0.3, while the contact angle value of AuNP-MIP plasmonic sensor surface was 76.3° ± 0.7. The reason for this decrease in the contact angle values was that the water-affinity (hydrophilic) property of the sensor surface had increased. The AuNP-MIP plasmonic sensor was relatively hydrophilic due to the added functional monomer and template molecule used in the preparation of the plasmonic sensor surface. In addition, the contact angle values of MIP and AuNP-NIP plasmonic sensor surfaces were measured as 77.9° ± 1.2 and 73.6° ± 1.1, respectively. The presence of AuNP increased the hydrophilic character of the plasmonic sensor surfaces.

The chemical structure analysis of AuNP-MIP, MIP, and AuNP-NIP plasmonic sensor surfaces was performed by FTIR spectroscopy. First, AuNP-MIP and MIP plasmonic sensors were evaluated by comparing FTIR spectra to determine whether AuNPs added to the structure were included in order to increase the plasmon signal response (Figure S3). Then, the FTIR spectra of AuNP-MIP and AuNP-NIP plasmonic sensors, as shown in Figure S4, were compared and evaluated in order to determine the imprinting efficiency. Accordingly, the N-H bonds seen in wavenumbers of 1664 cm^−1^ and 1587 cm^−1^, which became evident in the spectrum of the AuNP-MIP plasmonic sensor, indicate that the structure became integrated by coordinating with AuNP. The increase in the peak intensity of the C=O bond seen at the 1732 cm^−1^ wavenumber in the AuNP-MIP plasmonic sensor and the shift of the peak of the amide I bond seen at the 1670 cm^−1^ wavenumber in the MIP plasmonic sensor to the 1664 cm^−1^ wavenumber in the AuNP-MIP plasmonic sensor indicated that AuNP has been successfully included in the structure. The C=O bond seen at the 2938 cm^−1^ wave number and the peak of the C-N bond seen at the 1316 cm^−1^ wavenumber for the AuNP-MIP plasmonic sensor indicated that cortisol had been successfully integrated into the structure. In addition, the shift of the peak of the C=O bond seen at the 1732 cm^−1^ wavenumber in the spectrum of the AuNP-MIP plasmonic sensor to the 1729 cm^−1^ in the AuNP-NIP plasmonic sensor and a significant decrease in its intensity indicates that cortisol was involved in the structure [34]. The FTIR spectrum of blank cortisol was shown in Figure S5.

### 3.3. Kinetic Analyses with Plasmonic Sensors

**Figure 4:**
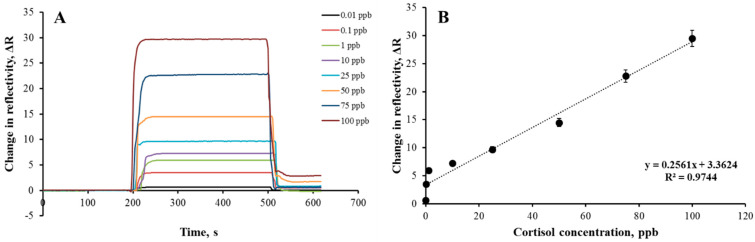
Real-time sensorgrams (**A**) and calibration graph (**B**) of AuNP-MIP plasmonic sensor at different concentrations of cortisol samples. Cortisol concentration: 0.01–100 ppb; adsorption solution: PBS-ethyl alcohol (50:50, *v*/*v*); interaction time: 10 min; flow rate: 0.2 mL/min; T: 25 °C.

In this study, AuNP-MIP, MIP, and AuNP-NIP plasmonic sensors were prepared and sensorgrams were obtained as a result of kinetic analyses. The equilibrium solution was passed first, then the adsorption and desorption solutions were passed through the sensor system, and these steps were repeated for each concentration. For real-time cortisol determination with AuNP-MIP plasmonic sensor, kinetic analyses were performed using cortisol samples prepared in the concentration range of 0.01–100 ppb. The sensorgrams of the sensor responses given by AuNP-MIP plasmonic sensor of cortisol solutions prepared at different concentrations were shown in Figure 4A and it was easily observed that the sensor response increased as the cortisol concentration increased. However, looking at the calibration graph obtained in Figure 4B, it can be concluded that the AuNP-MIP plasmonic sensor can detect cortisol with 97.44% accuracy in the range of 0.01–100 ppb cortisol concentration, with the equation y = 0.2561x + 3.3624. When the AuNP-MIP plasmonic sensor response for different cortisol concentrations were drawn, two different affinity behaviors were observed for the low and higher concentration levels separately. As shown in Figure S6, the regression coefficient value (R^2^ = 0.7818) for the low concentration range (0.01–1 ppb) was lower than the regression coefficient value (R^2^ = 0.9857) obtained for the higher concentration range (10–100 ppb) of cortisol. The recorded regression coefficient values confirmed that the AuNP-MIP plasmonic sensor had a higher affinity for the high concentration levels of cortisol, but in total for the whole concentration range, the obtained R^2^ value was sufficiently higher to explain the behavior of sensor responses. The limit of detection (LOD) and limit of quantity (LOQ) values of AuNP-MIP plasmonic sensor were calculated as 0.0082 ppb and 0.027 ppb, respectively.

From the kinetic analyses of different cortisol concentrations of AuNP-MIP plasmonic sensor, mathematical calculations were obtained to estimate the best-fitted isotherm model. Two different isotherm models (Langmuir and Freundlich) were applied to determine the type of interaction between the AuNP-MIP plasmonic sensor and the cortisol sample solution. Association and binding kinetic analyses were also obtained. According to the results, the theoretical ΔR_max_ value (6.991) calculated in the Scatchard analysis was quite close to the experimentally obtained ΔR_max_ value (7.270). Likewise, according to the binding analysis result, it was observed that cortisol binds to the AuNP-MIP plasmonic sensor surface with an accuracy of 98% (Figure S7). Furthermore, it was shown that the most suitable model for cortisol detection was the Langmuir adsorption isotherm model, which meant that the AuNP-MIP plasmonic sensor and cortisol binding was homogeneous, and the monolayer had a minimum lateral interaction. The theoretical ΔR_max_ value (6.916) calculated in the Langmuir adsorption isotherm model with the obtained constants was quite close to the experimentally obtained ΔR_max_ value (7.270). In addition, when the correlation coefficients obtained according to the mathematical calculations were compared, it was seen that the Langmuir adsorption isotherm model (R^2^ = 0.9999) had a higher correlation coefficient than the Freundlich adsorption isotherm model (R^2^ = 0.9082). The kinetic constants are summarized in Table S1.

### 3.4. Selectivity and Reusability Analyses

**Figure 5:**
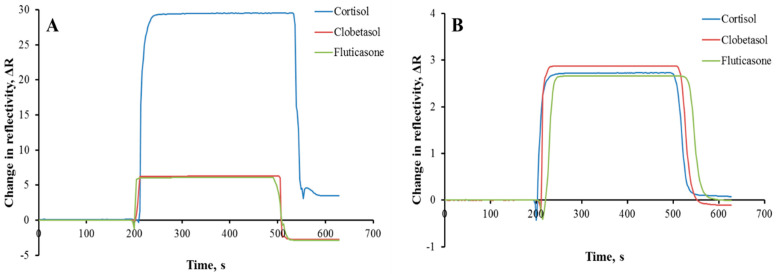
Selectivity sensorgrams of AuNP-MIP (**A**) and AuNP-NIP (**B**) plasmonic sensors. Cortisol concentration: 100 ppb; adsorption solution: PBS-ethyl alcohol (50:50, *v*/*v*); interaction time: 10 min; flow rate: 0.2 mL/min; T: 25 °C.

Within the scope of this study, a sensitive and selective plasmonic sensor was prepared for real-time cortisol determination at a low cost. For this purpose, AuNP was coordinated with MAH containing the amino acid histidine used as the imidazole group provider. The amino acid-based reusable AuNP-MIP plasmonic sensor could detect the cortisol at the ppb concentration level. Molecularly imprinted polymers are used to create a specific cavity and selective recognition sites for the template molecule in a polymeric structure. As shown in Figure 5A, competitive adsorption studies with fluticasone and clobetasol were performed to examine the selective determination of cortisol by the AuNP-MIP plasmonic sensor. For this purpose, clobetasol (100 ppb) and fluticasone (100 ppb) samples prepared separately were interacted with AuNP-MIP plasmonic sensor and non-specific interactions were observed to be relatively low.

In addition, the imprinting efficiency of AuNP-MIP plasmonic sensor was demonstrated with AuNP-NIP plasmonic sensor prepared without using cortisol. It was observed that the ΔR value decreased from 29.52 to 2.73 in the cortisol determination with the 100 ppb concentration made with AuNP-NIP plasmonic sensor (Figure 5B). Thus, it was observed that the AuNP-NIP plasmonic sensor did not interact significantly with cortisol, with the decrease in signal intensity. In addition, solutions prepared using fluticasone and clobetasol competitor agents interacted with AuNP-NIP plasmonic sensor. According to the results, it was observed that the AuNP-NIP plasmonic sensor gave non-specific low signals against competitive agents. The calculations of the selectivity (k) and relative selectivity (k′) coefficients are shown in Table 1. The AuNP-MIP plasmonic sensor was found to be 4.96 and 4.71 times more selective to the cortisol than the AuNP-NIP plasmonic sensor.

**Figure 6:**
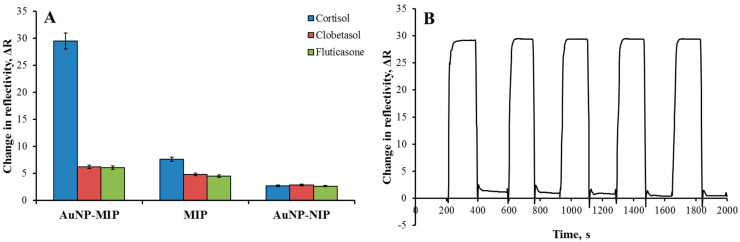
Selectivity comparison graph (**A**) and reusability sensograms (**B**) of AuNP-MIP plasmonic sensor. Cortisol concentration: 100 ppb; adsorption solution: PBS-ethyl alcohol (50:50, *v*/*v*); flow rate: 0.2 mL/min; T: 25 °C.

Moreover, the selectivity and signal enhancement performances of AuNP-MIP, MIP, and AuNP-NIP plasmonic sensors were obtained by interacting clobetasol (100 ppb) and fluticasone (100 ppb) samples and observed that the interactions of AuNP-NIP and MIP plasmonic sensors were less than the AuNP-MIP plasmonic sensor (Figure 6A). In particular, AuNP ranging in diameter from 5 to 40 nm has been widely used to enhance the response of SPR sensors [35]. Among all metal nanoparticles, gold and silver nanoparticles are particularly interesting because they have the strongest plasmonic interaction with light [36]. The signal enhancement by employing AuNP can be caused by different factors such as surface mass increasement due to AuNP and more molecules being attached to the AuNP than the planar gold surface; refractive index change by the particles; or electromagnetic field coupling between SPR and LSPR [37].

The reusability study of the AuNP-MIP plasmonic sensor was investigated by applying five repetitive equilibrium adsorption-desorption cycles of 100 ppb cortisol solution to the sensor system, and the sensor response was shown in Figure 6B. According to the results, the AuNP-MIP plasmonic sensor surface could be used repeatedly without any deterioration in the binding sites and binding capacity with any loss of performance.

### 3.5. Complex Environment Analyses

**Figure 7:**
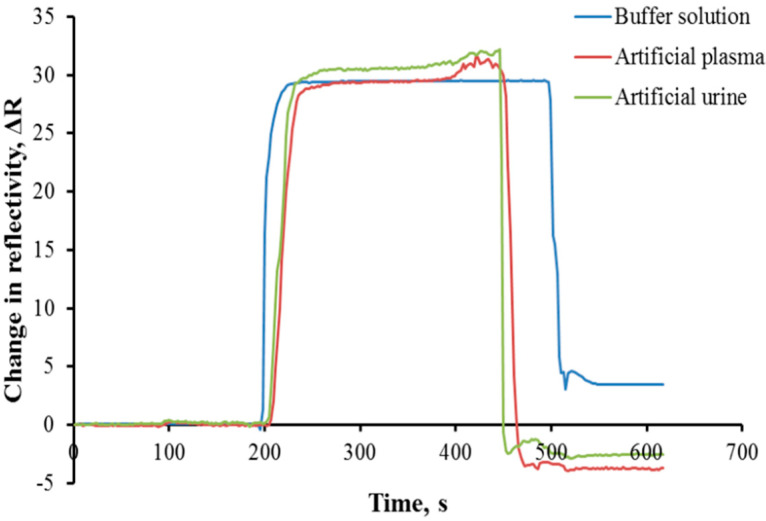
Real-time sensorgrams of AuNP-MIP plasmonic sensor for complex samples. Cortisol concentration: 100 ppb; adsorption solution: PBS-ethyl alcohol (50:50, *v*/*v*); interaction time: 10 min; flow rate: 0.2 mL/min; T: 25 °C.

In order to evaluate the AuNP-MIP plasmonic sensor for use in real samples, artificial plasma and artificial urine samples were prepared. As demonstrated in Figure 7, cortisol determination was performed from artificial plasma and artificial urine samples spiked with cortisol of a known concentration. The AuNP-MIP plasmonic sensor could detect cortisol not only in an aqueous solution, but also in complex environments such as artificial plasma and/or artificial urine. It was observed that other molecules in the same environment did not interfere with the binding sites of the AuNP-MIP plasmonic sensor and there was no loss in the detection performance.

The determination of the cortisol is very important for the human body. Many types of sensors have been developed for the determination of cortisol. Some of the reasons for the increase in studies on sensors in the last two decades are that they have different parameters such as a low detection limit, selectivity, reusability, and can be combined with other methods. In Table 2, the sensor responses consisting of using different sensor types for the determination of cortisol were compared. As reported in Table 2, there are some studies with better detection limits but known SPR-based sensors have superior advantages over traditional analytical techniques as they enable direct, label-free, real-time detections.

### 3.6. Validation Analyses

**Figure 8:**
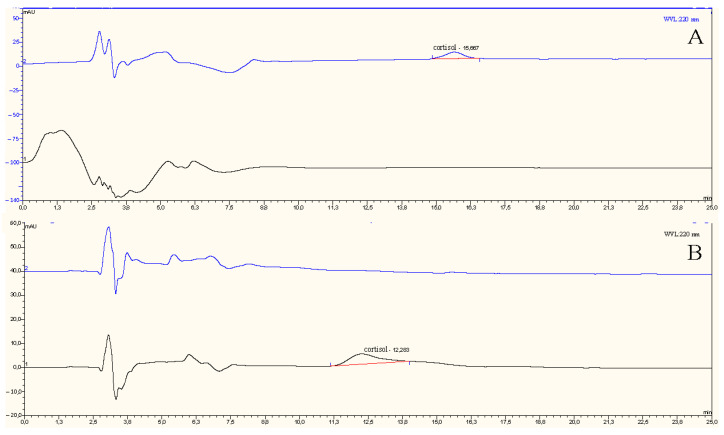
HPLC chromatograms of cortisol-spiked and unspiked artificial urine (**A**) and artificial plasma (**B**) samples.

The selective determination of cortisol in artificial plasma and artificial urine samples using AuNP-MIP plasmonic sensor was confirmed with HPLC system. For this purpose, cortisol solutions at concentrations of 1, 25, 50, 75, and 100 ppb were prepared and given to the HPLC system to create a calibration curve (Figure S8). Artificial plasma and artificial urine solutions containing cortisol were prepared by adding cortisol and were analyzed with a reversed-phase C18 column. The stock solution of cortisol was prepared in a mixture of 10 mM acetonitrile:methylalcohol:water (25:21:54) at a concentration of 1 mg/mL. The solutions used in the calibration were prepared by diluting the stock solution with a mixture. The amount of cortisol was determined by the isocratic elution system with analyses made at 220 nm using HPLC system. It was determined that the amount of cortisol in artificial plasma and artificial urine solutions spiked with 100 ppb cortisol was directly proportional to the results predicted by the AuNP-MIP plasmonic sensor. The chromatograms of artificial urine with 100 ppb cortisol are shown in Figure 8A and artificial plasma in Figure 8B. The cortisol concentration level was determined from the obtained area (0.001) using the calibration curve for the 0.01 ppb cortisol concentration, which was the lowest concentration level of cortisol applied to the AuNP-MIP plasmonic sensor.

## 4. Conclusions and Discussion

In this study, a cortisol-imprinted plasmonic sensor modified with gold nanoparticles was prepared, and then real-time and sensitive cortisol detection was carried out both from an aqueous solution and a complex environment such as artificial plasma and artificial urine without applying any extra complex processes such as ligand immobilization or using any spacer arms. Moreover, the amino acid-based functional monomer containing histidine groups was used as the provider of the complexing agent for AuNP and cortisol molecules in one mode. The AuNP-MIP plasmonic sensor that contained cortisol-specific recognition sites was prepared to detect cortisol selectively, and gold nanoparticles were added to the medium in order to reduce the detection limit of cortisol and increase the sensitivity of the plasmonic sensor signal response. Other advantages of this plasmonic sensor were its thermal and chemical stability, low-cost, long shelf-life, and also more sensitive cortisol detection several times over without extra labeling alternatives to the natural receptors.

In a nutshell, different characterization experiments showed that the imprinted polymer was successfully and homogeneously synthesized on the gold surfaces of the plasmonic sensor. Then, kinetic analyses results depicted that the AuNP-MIP plasmonic sensor was able to detect cortisol in real-time with a correlation coefficient of 0.9744 for the 0.01–100 ppb concentration range. Accordingly, the AuNP-MIP plasmonic sensor was found to be 4.96 and 4.71 times more selective to the cortisol than the AuNP-NIP plasmonic sensor for the clobetasol and fluticasone molecules, respectively. The signal enhancement effect was confirmed by evaluating the signal responses of the MIP plasmonic sensor to the cortisol. The signal enhancement factor recorded as 5.6 confirmed the effect of gold nanoparticles on the sensitivity by enhancing the signal response of the plasmonic sensor. Furthermore, it was concluded that the AuNP-MIP plasmonic sensor was able to detect cortisol without any loss of performance in five consecutive reusability analyses. The AuNP-MIP plasmonic sensor could detect cortisol not only in aqueous solutions, but also in complex environments such as artificial plasma and/or artificial urine to evaluate the matrix effect. Validation experiments performed with HPLC studies confirmed the detection ability of the AuNP-MIP plasmonic sensor for the cortisol. At last, it can be concluded that the AuNP-MIP plasmonic sensor as developed is a good proof-of-concept for the future and development of field sensors for use in medical applications.

## Figures and Tables

**Table 1:** Selectivity (k) and relative selectivity (k′) coefficients of plasmonic sensors.

AuNP-MIP			AuNP-NIP		
	ΔR	k	ΔR	k	k′
Cortisol	29.52	-	2.73	-	-
Clobetasol	6.26	4.72	2.87	0.95	4.96
Fluticasone	6.11	4.83	2.66	1.03	4.71

**Table 2:** Comparison of studies for cortisol detection.

Reference	Sensor Type	Detection Range	Detection Limit	Real Sample
[3]	Immunosensor	0.1–10 ng/mL	-	Saliva
[18]	Optical	1.5–10 ng/mL	1.0 ng/mL	Saliva
[38]	Optical	9–132 μg/L	3 μg/L	Saliva
[39]	Electrochemical	1 pg/mL–10 ng/mL	0.87 pg/ mL	Saliva
[40]	Electrochemical	10–200 ng/mL	1 pg/mL	Human and Artificial Sweat
[41]	Electrochemical	1.81–36.2 ng/mL	18 fg/mL	Saliva
[42]	Electrochemical	3.6–3624 ng/mL	-	Sweat
[43]	Electrochemical	0.5–500 ng/mL	0.5 ng/mL	Sweat
[44]	Electrochemical	0.003–36 ng/mL	10 pg/mL	-
[45]	Electrochemical	0.003–181 ng/mL	0.36 pg/mL	-
[46]	Electrochemical	0.36 pg/mL–3624 ng/mL	3624 ng/mL	-
[47]	Immunosensor	10.8 pg/mL–0.36 μg/mL	10.8 pg/mL	Serum
[48]	Electrochemical	1–100 ag/mL	2 ag/mL	Urine
[49]	Electrochemical	0.001–50 ng/mL	1.03 pg/mL	Artificial Salivia
[50]	Electrochemical	0.1–1000 ng/mL	0.05 ng/mL	Serum
[51]	Electrochemical	0.18–72 ng/mL	40 pg/mL	Saliva
[52]	Electrochemical	3.6–28.9 μg/mL	7.4 μg/mL	Serum
This work	Optical	0.01–100 ppb (ng/mL)	0.0082 ppb (ng/mL)	Artificial Plasma and Urine

## Supplementary Materials

The following supporting information can be downloaded at: https://www.mdpi.com/article/10.3390/bios12070482/s1, Figure S1: Direct attachment of vinyl group onto gold chip surface (A) and formation of MAH-AuNP-cortisol pre-complex (B); Figure S2: FTIR spectra of MAH, MAH-AuNP and MAH-AuNP-cortisol pre-complex; Figure S3: FTIR spectra of AuNP-MIP and MIP plasmonic sensors; Figure S4: FTIR spectra of AuNP-MIP and AuNP-NIP plasmonic sensors; Figure S5: FTIR spectra of cortisol; Figure S6: AuNP-MIP plasmonic sensor response for two different concentration ranges (0.01–1 ppb and 10–100 ppb) of cortisol; Figure S7: Scatchard (A), binding (B) kinetic analysis, Langmuir (C) and Freundlich (D) isotherm models; Figure S8: Calibration curve used for HPLC analyses of cortisol. Table S1: Kinetic constants; Table S2: Adsorption isotherm model constants.
