# Supplementary material for: Selective Amplification of Plasmonic Sensor Signal for Cortisol Detection Using Gold Nanoparticles
Source: Biosensors (Basel). 2022 Jul 1;12(7):482. doi: 10.3390/bios12070482 (PMC9313393; doi:10.3390/bios12070482)
Supplement: Supplementary file 1 [file biosensors-12-00482-s001.zip › biosensors-1746187-supplementary.pdf]

Selective Amplification of Plasmonic Sensor Signal for Cortisol Detection using Gold Nanoparticles

Gaye Ezgi Yılmaz[1], Yeşeren Saylan[1], Ilgım Göktürk[1], Fatma Yılmaz[2], Adil Denizli[1*]

[1]Hacettepe University, Department of Chemistry, Beytepe, Ankara, Turkey

[2] Bolu Abant Izzet Baysal University, Department of Chemistry Technology, Bolu, Turkey

Supplementary document

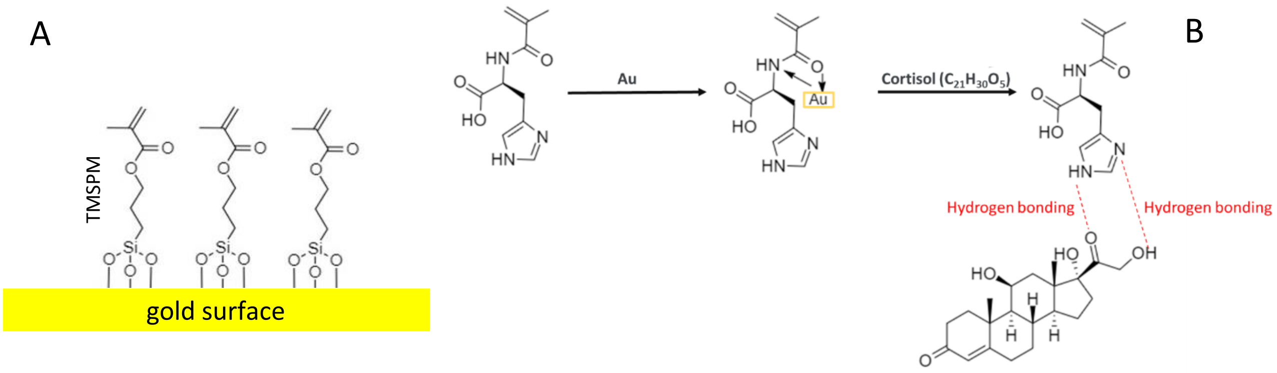

Figure S1. Direct attachment of vinyl group onto gold chip surface (A) and formation of MAH-AuNP-cortisol pre-complex (B).

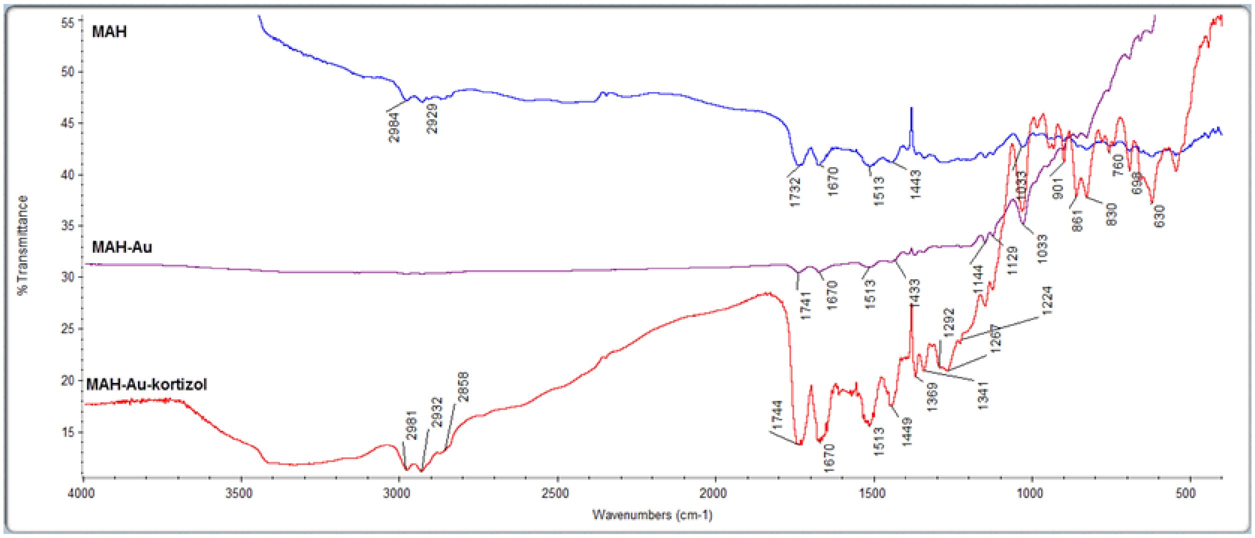

Figure S2. FTIR spectra of MAH, MAH-AuNP and MAH-AuNP-cortisol pre-complex.

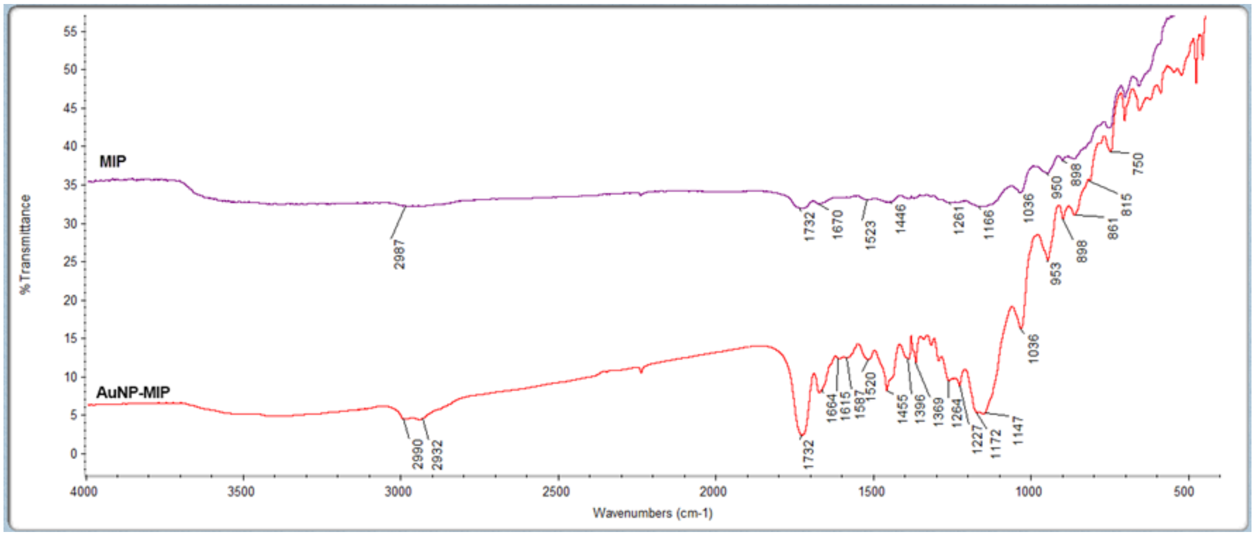

Figure S3. FTIR spectra of AuNP-MIP and MIP plasmonic sensors.

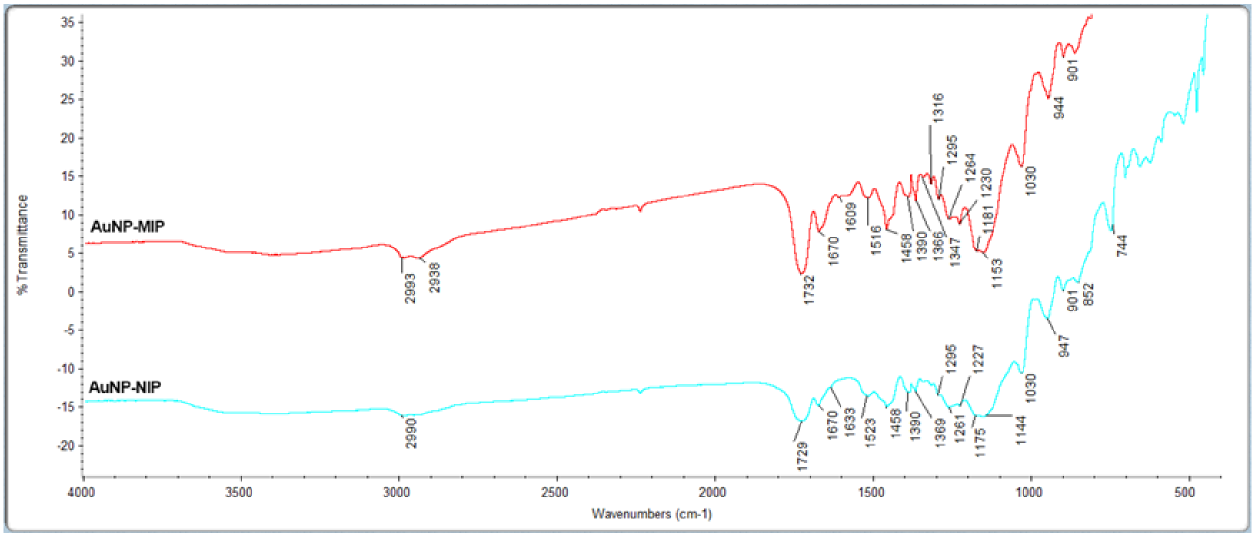

Figure S4. FTIR spectra of AuNP-MIP and AuNP-NIP plasmonic sensors.

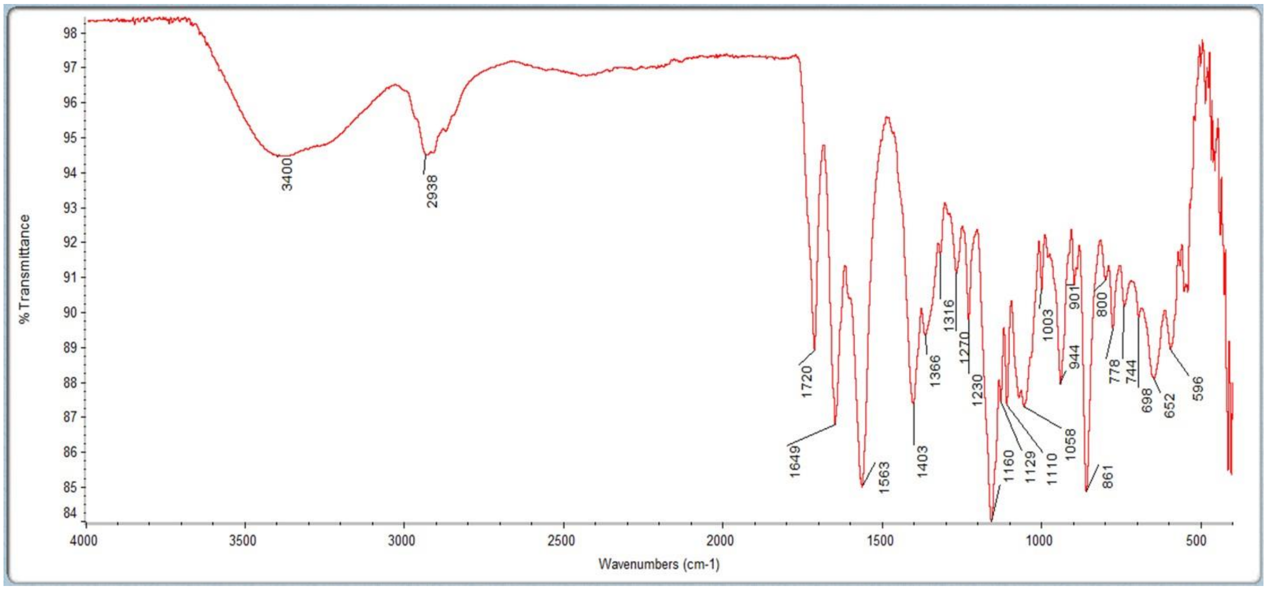

Figure S5. FTIR spectra of cortisol.

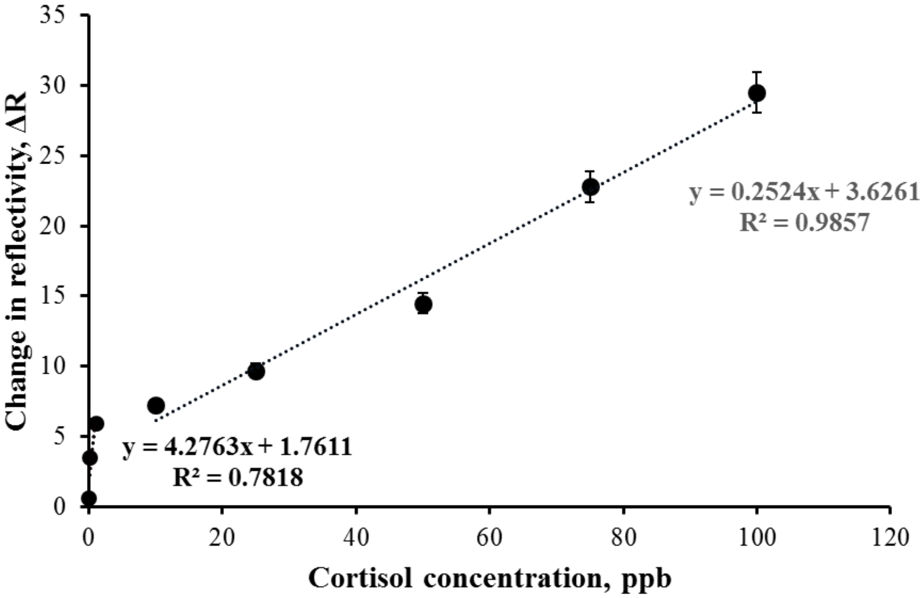

Figure S6. AuNP-MIP plasmonic sensor response for two different concentration ranges (0.01-1 ppb and 10-100 ppb) of cortisol.

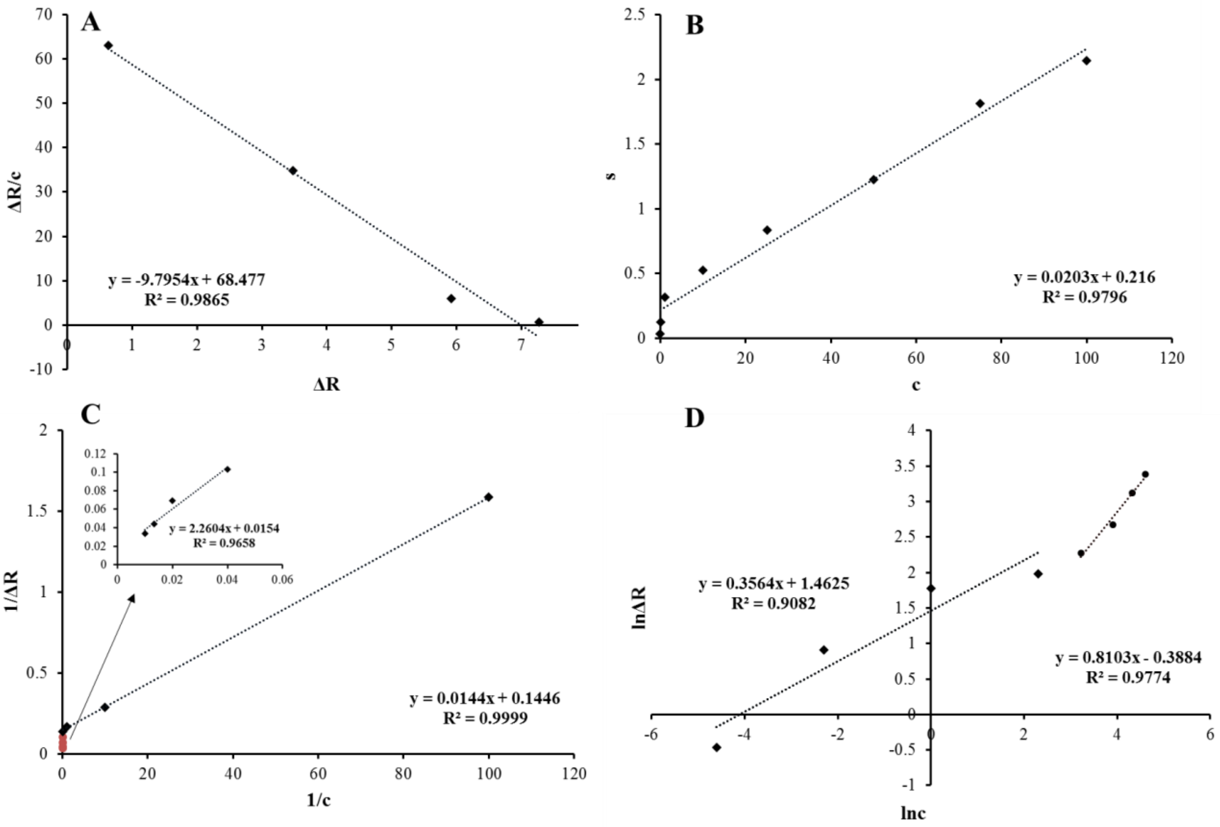

Figure S7. Scatchard (A), binding (B) kinetic analysis, Langmuir (C) and Freundlich (D) isotherm models.

Table S1. Kinetic constants.

**Association Analysis (Scatchard)**		**Binding Kinetic Analysis**	
ΔR_{max}	6.991	k_a	0.0203
K_A	9.795	k_d	0.216
K_D	0.102	K_A	0.094
R^2	0.987	K_D	10.64
		R^2	0.980

Table S2. Adsorption isotherm model constants.

Langmuir		Freundlich	
ΔR_{max}	6.916	ΔR_{max}	4.32
K_A	480.3	1/n	0.356
K_D	0.002	R^2	0.908
R^2	0.999		

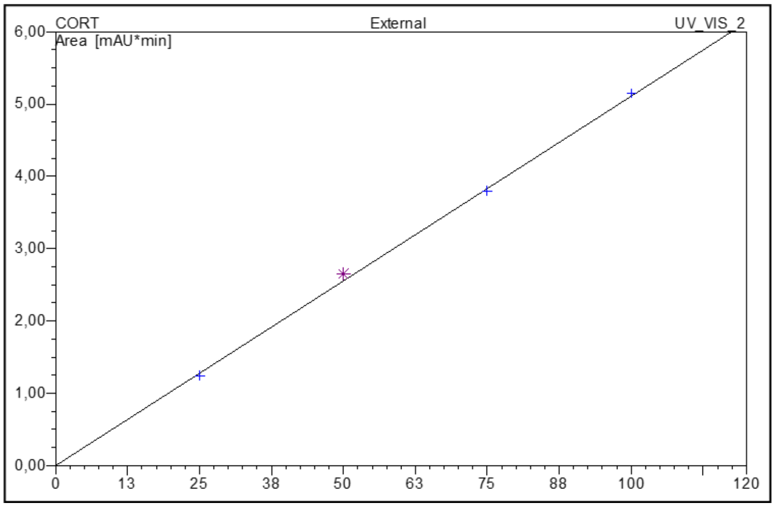

Figure S8. Calibration curve used for HPLC analyses of cortisol.
